# Mechanisms, prevention, and management of aminoglycoside-induced hearing loss in neonates and children: a translational review

**DOI:** 10.3389/fphar.2026.1823729

**Published:** 2026-06-10

**Authors:** Sílvia M. Illamola, Catherine M. Sherwin, Lauren C. Ferguson, Angela K. Birnbaum, Ravindhra G. Elluru, Janelle D. Vaughns

**Affiliations:** 1 Department of Experimental and Clinical Pharmacology, College of Pharmacy, University of Minnesota, Minneapolis, MN, United States; 2 Internal Medicine, UWA Medical School, The University of Western Australia, Perth, WA, Australia; 3 Differentia Biotech Ltd., South San Francisco, CA, United States; 4 Department of Pharmacology and Toxicology, Wright State University Boonshoft School of Medicine, Dayton, OH, United States; 5 Department of Emergency Medicine, The University of Texas Southwestern Medical Center, Dallas, TX, United States; 6 Center for Clinical and Cognitive Neuropharmacology, College of Pharmacy, University of Minnesota, Minneapolis, MN, United States; 7 Department of Medical Laboratory Sciences, College of Pharmacy, University of Minnesota, Minneapolis, MN, United States; 8 Pediatric Otolaryngology, Peyton Manning Children’s Hospital, Indianapolis, IN, United States; 9 Department of Anesthesiology, Pain and Perioperative Medicine, Children’s National Hospital, GW School of Medicine and Health Sciences, The George Washington University, Washington, DC, United States; 10 Division of Clinical Pharmacology, Children’s National Hospital, GW School of Medicine and Health Sciences, The George Washington University, Washington, DC, United States

**Keywords:** aminoglycosides, genetic susceptibility, hearing loss, MT-RNR1 mutation, ototoxicity, pediatric, therapeutic drug monitoring

## Abstract

**Objective:**

Aminoglycoside antibiotics remain essential for treating serious neonatal and pediatric infections, yet carry a well-documented risk of permanent auditory and vestibular toxicity. This review examines the pharmacological mechanisms of ototoxicity in pediatric populations, identifies those at highest risk, and assesses current prevention, monitoring, and management strategies.

**Methods:**

PubMed, EMBASE, Cochrane Library, Web of Science, and CINAHL were searched for relevant literature from 2000 to 2025. The primary focus was pediatric populations, though mechanistic and translational pharmacology work from other age groups was included.

**Results:**

The ototoxicity pathway is increasingly well characterized: aminoglycosides accumulate in cochlear hair cells via mechanoelectrical transduction channels, disrupt mitochondrial function, trigger oxidative stress, and cause cell death through apoptotic, necroptotic, and ferroptotic mechanisms. Susceptibility varies substantially. Patients carrying the MT-RNR1 m.1555A>G pharmacogenomic variant face a markedly elevated risk of profound hearing loss even with a single standard course. Preterm neonates are at higher risk than older children owing to renal immaturity, altered volume of distribution, and incomplete blood-labyrinth barrier development. Co-administration of loop diuretics and vancomycin further amplifies ototoxic risk. Extended-interval dosing is associated with equivalent efficacy and reduced nephrotoxicity, with a non-significant trend toward lower ototoxicity in pooled analyses. Point-of-care genetic screening allows identification of high-risk patients before the first dose, though debate continues over universal versus targeted implementation. Model-informed dosing approaches, including Bayesian forecasting and AUC-targeted monitoring, offer individualized pharmacokinetic optimization but remain underutilized. Antimicrobial stewardship and minimizing concomitant ototoxin exposure are complementary strategies. When ototoxicity occurs, early audiological and vestibular identification enables timely intervention through hearing aid fitting, cochlear implantation, vestibular rehabilitation, and family-centered support, though vestibular ototoxicity remains widely under-recognized in pediatric populations.

**Conclusion:**

Evidence-based interventions to reduce aminoglycoside ototoxicity in children exist, including pharmacogenomic screening and dosing optimization, as well as structured monitoring and rehabilitation. However, a persistent gap remains between available evidence and routine clinical implementation. Key research priorities include pediatric otoprotective trials, validated cochlear injury biomarkers, and implementation strategies for diverse healthcare settings. Given the permanence of aminoglycoside-induced ototoxic injury and its downstream effects on speech, language, and developmental outcomes, closing this gap represents an urgent clinical priority.

## Introduction

Thirty-four million children worldwide have hearing loss, with an estimated 60% of these cases potentially preventable (WHO, 2024). Aminoglycosides account for a meaningful fraction of preventable childhood hearing loss (Lanvers-Kaminsky and Ciarimboli, 2017; Diepstraten et al., 2021; Saunders et al., 2009). Aminoglycosides remain a cornerstone of empirical therapy and are among the most frequently prescribed antibiotics for suspected serious gram-negative infections in newborns (Ting et al., 2016; Cantey et al., 2015; Tamma et al., 2012). Yet they damage the inner ear in ways that cannot be undone (Forge and Schacht, 2000; Huth et al., 2011). Clinicians face this trade-off daily. The consequences are not trivial: unlike aminoglycoside nephrotoxicity, which typically resolves after drug discontinuation, ototoxicity produces permanent sensorineural hearing loss (Diepstraten et al., 2021; Schacht et al., 2012; Forge and Li, 2000). The scale of exposure compounds the concern, and the patients receiving aminoglycosides are precisely those most vulnerable to their toxic effects. Preterm and critically ill neonates are at particular risk, for reasons discussed in the Neonatal Vulnerability section below (Zimmerman and Lahav, 2013; Suzuki et al., 1998).

Most of the existing literature addresses adults, focuses narrowly on the mechanism, or ignores the developmental pharmacology that makes pediatric patients different (Diepstraten et al., 2021). Where pediatric data do exist, they are fragmented across studies that vary widely in monitoring intensity, diagnostic thresholds, and population characteristics, making synthesis difficult and clinical translation uncertain (Lanvers-Kaminsky and Ciarimboli, 2017; Diepstraten et al., 2021; Garinis et al., 2017a; Jacqz-Aigrain et al., 2012). This review brings these threads together, aiming to provide practical, age-specific guidance for clinicians managing aminoglycoside therapy in neonates and young children, integrating mechanistic, pharmacokinetic, and clinical evidence across developmental stages -- distinctions that adult-derived guidelines consistently overlook (Table 1).

**TABLE 1:** Characterization of evidence included in this narrative review.

Evidence category	Types of studies	Evidence level*	Strengths	Limitations
Mechanistic studies	*In vitro* cochlear models, animal studies, and molecular biology investigations	Preclinical	Provides a detailed understanding of cellular pathways and temporal progression	Limited direct translation to clinical settings; species differences
Genetic studies	Mitochondrial DNA analyses, genome-wide association studies, and pharmacogenetic investigations	Preclinical/Observational clinical	Identifies specific risk factors; allows targeted screening	Variable penetrance; incomplete understanding of gene–environment interactions
Clinical observational studies	Cohort studies, case-control studies, retrospective analyses	Observational clinical	Documents real-world incidence, risk factors, and outcomes in pediatric populations	Confounding variables; heterogeneity in definitions and outcome measures
Interventional research	Clinical trials of protective agents, monitoring protocols, and dosing strategies	Randomized clinical/Observational clinical	Provides direct evidence for preventive approaches	Limited randomized controlled trials in pediatric populations due to ethical constraints; ototoxicity endpoints are inconsistently measured across studies
Guidelines and expert opinion	Consensus statements, professional society guidelines	Guideline consensus	Synthesizes evidence into practical recommendations	May lag behind the most recent evidence; varies by region

* Evidence Level categories: Preclinical = *in vitro* and/or animal model data; Observational clinical = cohort, case-control, or retrospective human studies; Randomized clinical = randomized controlled trials; Guideline consensus = professional society consensus statements or guidelines. Where a category spans multiple evidence levels, both are listed.

## Background

### Epidemiology and clinical burden

Reported ototoxicity rates range from 2% to 63% across pediatric populations, reflecting differences in monitoring intensity, diagnostic thresholds, follow-up duration, and population risk profiles (Lanvers-Kaminsky and Ciarimboli, 2017; Diepstraten et al., 2021; Garinis et al., 2017a). Genetic susceptibility, particularly the MT-RNR1 m.1555A>G variant, substantially elevates individual risk even at standard therapeutic doses (McDermott et al., 2022a; McDermott et al., 2022b), as discussed in the Genetic Susceptibility section below. In the United States, the CDC National Vital Statistics System, which collects data from state birth certificate records, indicates that approximately 9% of newborns are admitted to neonatal intensive care units annually (8.7%–9.6% between 2016 and 2021) (Martin and Osterman, 2025), with rates varying by maternal age and other factors (Gamber et al., 2024). Aminoglycosides, especially gentamicin, are among the most commonly prescribed antibiotics in NICUs (Ting et al., 2016; Cantey et al., 2015) due to their efficacy against gram-negative pathogens and their synergistic activity with β-lactams (Tamma et al., 2012).

**FIGURE 1:**
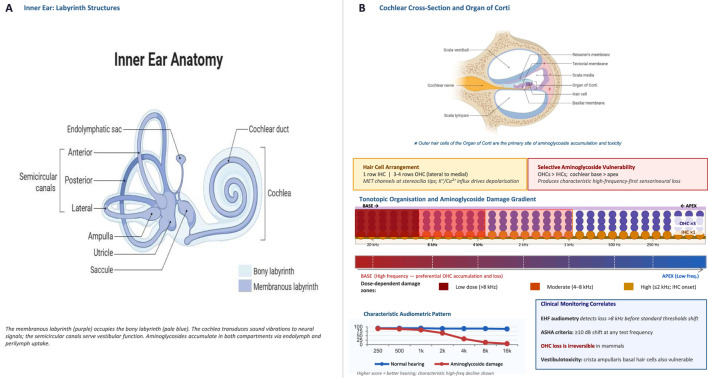
Anatomy of the Inner Ear and Organ of Corti Relevant to Aminoglycoside Ototoxicity. **(A)** The membranous labyrinth (purple) is housed within the bony labyrinth (pale blue) and comprises the cochlea, semicircular canals, utricle, saccule, and endolymphatic sac. The cochlea transduces sound-induced vibrations into neural signals transmitted via the cochlear nerve; the semicircular canals and otolithic organs (utricle and saccule) subserve vestibular function. Both the endolymph and perilymph compartments accumulate aminoglycosides following systemic administration. **(B)** Cross-section through one cochlear turn showing the three fluid-filled scalae (scala vestibuli, scala media, scala tympani), Reissner’s membrane, the tectorial membrane, the basilar membrane, and the organ of Corti containing the mechanosensory hair cells. A single row of IHCs and three rows of OHCs are arranged along the full length of the basilar membrane. MET channels at the tips of the stereocilia admit K^+^ and Ca^2+^ upon deflection, depolarizing the hair cell and initiating afferent signaling. The unrolled basilar membrane schematic (center) illustrates the tonotopic arrangement of three OHC rows and one IHC row (amber, ×1) from the high-frequency cochlear base to the low-frequency apex, with audiometric frequency positions mapped to cochlear location using the Greenwood function. Colour coding of the OHC rows reflects cumulative aminoglycoside-induced hair cell loss by exposure zone: dark red (>8 kHz; basal outer hair cells, lost at low cumulative doses), orange (4–8 kHz; moderate cumulative doses), and amber (≤2 kHz; high cumulative doses, with onset of IHC involvement). The representative audiogram (lower left) illustrates the characteristic high-frequency-first pattern of aminoglycoside-induced hearing threshold elevation compared with normal hearing. OHC loss in mammals is irreversible. Aminoglycoside-induced vestibulotoxicity additionally affects hair cells of the crista ampullaris at the base of each semicircular canal. Abbreviations: IHC, inner hair cell; MET, mechanoelectrical transduction; OHC, outer hair cell; SCC, semicircular canal. Created in BioRender. Bitner-Glindzicz et al. (2009) https://BioRender.com/d58o4vz.

The inner ear is one of the three anatomical parts of the ear, consisting of the cochlea and the vestibular apparatus (semicircular canals, utricle, and saccule), which together constitute the membranous labyrinth, a fluid-filled (endolymph) system of sensory structures (Bruss and Shohet, 2026). Within the cochlea, the organ of Corti contains two functionally distinct populations of sensory cells: a single row of inner hair cells, which serve as the primary afferent transducers of sound, and three rows of outer hair cells, which amplify basilar membrane motion through electromotility (Forge and Schacht, 2000; Schacht et al., 2012). The vestibular apparatus contains separate mechanosensory receptors, Type I and Type II vestibular hair cells, which detect angular and linear acceleration (Schacht et al., 2012). The anatomical basis for aminoglycoside ototoxicity centers on the cochlea and vestibular organs (Figure 1) (Forge and Schacht, 2000; Schacht et al., 2012). Outer hair cells are especially vulnerable, and damage proceeds in a characteristic base-to-apex gradient, affecting high-frequency hearing first (Huth et al., 2011). This tonotopic pattern reflects both increased expression of MET channels in basal hair cells (Pan et al., 2018; Alharazneh et al., 2011) and their higher metabolic demands (Sha and Schacht, 1999). Type I vestibular hair cells show similar susceptibility (Forge and Li, 2000). Mammalian cochlear hair cells cannot regenerate (Forge and Li, 2000), and damage may progress even after treatment cessation due to persistent drug retention (Hailey et al., 2017) and ongoing oxidative stress (Sha and Schacht, 1999). Gentamicin primarily causes vestibular damage, while amikacin, kanamycin, and neomycin mainly affect the cochlea (Forge and Schacht, 2000; Huth et al., 2011; Selimoglu, 2007). These agent-specific toxicity patterns continue to inform drug selection in clinical practice (Forge and Schacht, 2000; McDermott et al., 2022b). Economic analyses estimate lifetime costs exceeding $1 million per affected individual when accounting for educational support, lost productivity, and healthcare utilization (Mohr et al., 2000). More recent modeling confirms that the societal economic burden remains substantial, with annual US costs estimated at $37 billion (Cejas et al., 2024).

### Neonatal Vulnerability

Age makes a significant difference. Neonates are especially vulnerable due to renal immaturity, altered volume of distribution, and incomplete blood-labyrinth barrier development, which together increase both drug accumulation and cochlear susceptibility. NICU infants have approximately 10-fold higher rates of hearing loss compared to well-baby nursery populations (2%–4% vs. 0.1%–0.3%), with prematurity and low birth weight consistently identified as independent risk factors (Zimmerman and Lahav, 2013; Al-Ani, 2023). The pharmacokinetic and physiological basis for this vulnerability is discussed in detail in the Age-Related Vulnerabilities section below.

## Literature search strategy

This narrative review is based on a structured literature search of PubMed, EMBASE, Cochrane Library, Web of Science, and CINAHL for English-language articles published between January 2000 and March 2025. Search terms included “aminoglycosides,” “ototoxicity,” “hearing loss,” “cochleotoxicity,” “vestibulotoxicity,” “vestibular,” “pediatric,” “neonate,” “infant,” “pharmacogenomics,” “MT-RNR1,” “mitochondrial,” and “therapeutic drug monitoring,” used in various combinations. Seminal publications predating the search window were included if they provided foundational mechanistic, pharmacological, or historical context that was not superseded by subsequent work.

All retrieved records were imported into EndNote reference management software (n = 273). Duplicate records were identified and removed using EndNote (n = 34). The remaining records (n = 239) were screened based on title and abstract, followed by iterative full-text review. We prioritized studies addressing pediatric populations and translational mechanistic research and examined the reference lists of included articles for additional relevant publications. Grey literature, conference abstracts, and non-English-language publications were not systematically included.

Studies were included based on relevance to predefined thematic areas, including mechanistic pathways of ototoxicity, pharmacokinetic and pharmacodynamic considerations, genetic susceptibility, and clinical outcomes. Studies were excluded if they did not address aminoglycoside-associated ototoxicity, lacked relevance to pediatric or neonatal populations, did not include clinically or mechanistically relevant outcomes, or were non-primary reports (e.g., editorials or conference abstracts without sufficient detail).

Age categories followed International Council for Harmonisation (ICH) E11 (R1) guidelines (International Council for Harmonisation of Technical Requirements for Pharmaceuticals for Human Use ICH, 2017): neonates (0–27 days), infants (28 days–23 months), children (2–11 years), and adolescents (12–18 years). We further stratified neonates by gestational age into preterm (<37 weeks) and term (≥37 weeks) subgroups to reflect the distinct pharmacokinetic and maturational considerations relevant to aminoglycoside disposition in this population.

**FIGURE 2:**
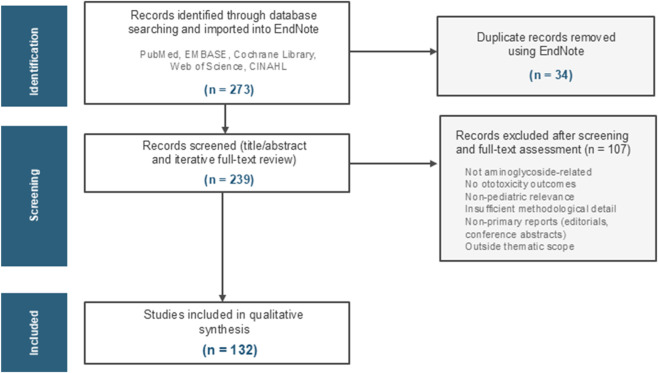
Adapted literature search and study selection workflow. A total of 273 records were identified through database searching of PubMed, EMBASE, Cochrane Library, Web of Science, and CINAHL, and imported into EndNote reference management software. Duplicate records were removed (n = 34), and the remaining records (n = 239) were screened by title and abstract followed by iterative full-text review. Records were excluded if they did not address aminoglycoside-associated ototoxicity, lacked relevance to pediatric or neonatal populations, did not include clinically or mechanistically relevant outcomes, had insufficient methodological detail to support inclusion, or were non-primary reports (editorials or conference abstracts). A total of 132 studies were included in the final qualitative synthesis. Because this review was conducted as a structured narrative review, screening and eligibility assessment were not conducted as discrete, prospectively recorded stages; accordingly, stage-specific record counts are not reported and the diagram reflects the overall selection process rather than sequential PRISMA-compliant stages. Record counts are reported for transparency rather than as reproducible PRISMA-compliant outputs. Exclusion reasons are presented as categories without individual counts, consistent with the iterative methodology described in the Literature Search Strategy section. Figure adapted from the PRISMA 2020 framework (Page et al., 2021).

This synthesis integrates mechanistic, clinical, and translational evidence without formal systematic review methodology or Preferred Reporting Items for Systematic Reviews and Meta-Analyses (PRISMA) reporting. Screening and eligibility assessment were conducted iteratively rather than as discrete, prospectively recorded stages; accordingly, stage-specific counts are not reported. Given the breadth of the topic and clinical heterogeneity in study designs, ototoxicity definitions, and audiometric endpoints across the literature, formal meta-analysis was not attempted. A total of 132 studies were included in the final synthesis. An adapted literature search and study selection workflow is provided to enhance transparency (Figure 2).

## Mechanisms of aminoglycoside-induced ototoxicity

### Cellular entry and accumulation

**FIGURE 3:**
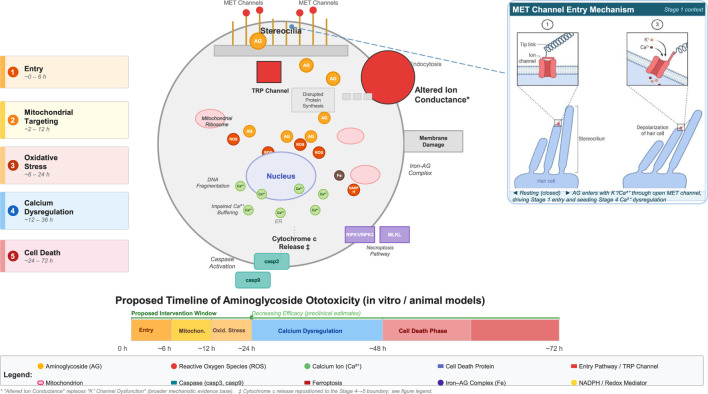
Cellular mechanisms and proposed timeline of aminoglycoside ototoxicity in cochlear outer hair cells. The main schematic depicts a stylised outer hair cell with five temporally ordered mechanistic stages, each color-coded and labeled on the left margin with approximate time windows derived from *in vitro* cochlear explant and *in vivo* rodent models. (1) Entry (∼0–6 h): aminoglycosides (AGs) enter outer hair cells primarily through mechanoelectrical transduction (MET) channels at stereocilia tips and via a secondary endocytotic route; AG interaction with TRP channels produces altered ion conductance,* disrupting K^+^ and Ca^2+^ flux and impairing mitochondrial ribosomal protein synthesis. (2) Mitochondrial Targeting (∼2–12 h): AGs accumulate in mitochondria and bind the 12S rRNA of the mitochondrial ribosome, causing translational errors and impaired oxidative phosphorylation, with consequent early ROS generation. (3) Oxidative Stress (∼6–24 h): iron–AG complexes catalyze Fenton-type free radical production; NADPH oxidase activation amplifies the oxidative burden; lipid peroxidation produces membrane damage and nuclear DNA fragmentation. (4) Calcium Dysregulation (∼12–36 h): oxidative injury to the endoplasmic reticulum (ER) impairs Ca^2+^ sequestration, producing pathological cytosolic Ca^2+^ elevation; sustained Ca^2+^ overload triggers cytochrome c release,‡ committing the cell to intrinsic apoptosis. (5) Cell Death (∼24–72 h): hair cell demise proceeds via three converging pathways -- intrinsic apoptosis (casp9/casp3 activation), RIPK1/RIPK3/MLKL-dependent necroptosis, and iron-dependent ferroptosis driven by unrestricted lipid peroxidation. The inset (upper right) illustrates the MET channel entry mechanism at the stereocilium tip. Panel 1 (left) shows the resting/closed state: the tip link is intact, the MET channel is closed, and no cation flux or AG entry occurs. Panel 3 (right) shows the open/depolarized state: stereociliary bundle deflection tensions the tip link, mechanically gating the MET channel open; K^+^, Ca^2+^, and AGs enter through the open pore driven by the endocochlear potential, constituting the principal Stage 1 entry mechanism; the resulting Ca^2+^ influx also seeds the dysregulation cascade of Stage 4. Panel 2 of the original MET transduction diagram (mechanical displacement) was omitted as it describes the normal auditory stimulus rather than the drug entry mechanism. The dashed connector links the inset to the MET channel zone of the main schematic. The horizontal timeline bar (base of figure) depicts the five colour-matched stage segments across the ∼72-h progression. The solid green bar (∼0–24 h) demarcates the proposed intervention window within which preclinical otoprotective strategies demonstrate maximal efficacy; the pale green zone (∼24–72 h) reflects progressively declining otoprotective efficacy as cell death commitment becomes irreversible. All timeframes are approximate and should not be extrapolated to clinical contexts, in which pharmacokinetic variability, patient age, renal function, aminoglycoside compound, and concurrent ototoxic exposures substantially modify the ototoxicity timeline. All depicted interventions are preclinical and have not been validated in randomised clinical trials.* “Altered Ion Conductance” replaces the previously used term “K^+^ Channel Dysfunction” to reflect mechanistic evidence implicating TRP channels and non-selective cation conductances in addition to K^+^-selective pathways in early AG-mediated electrophysiological disruption. ‡ Cytochrome c release is positioned at the Stage 4→5 boundary to reflect the temporal sequence observed in vitro models, wherein mitochondrial outer membrane permeabilization follows sustained Ca^2+^ dysregulation. Abbreviations: AG, aminoglycoside; Ca^2+^, calcium ion; casp3, caspase-3; casp9, caspase-9; ER, endoplasmic reticulum; Fe–AG, iron–aminoglycoside complex; MET, mechanoelectrical transduction; MLKL, mixed lineage kinase domain-like protein; NADPH, nicotinamide adenine dinucleotide phosphate (reduced); OHC, outer hair cell; RIPK1, receptor-interacting protein kinase 1; RIPK3, receptor-interacting protein kinase 3; ROS, reactive oxygen species; TRP, transient receptor potential. Created in BioRender. Sherwin C. (2026) https://BioRender.com/265z8w8.

Figure 3, Table 2 summarize the mechanistic cascade from initial drug uptake to terminal hair cell loss. Aminoglycosides enter outer hair cells principally through apical MET channels and, to a lesser extent, via megalin-mediated endocytosis (Huth et al., 2011; Pan et al., 2018; Alharazneh et al., 2011). Once internalized, the drug accumulates to concentrations several-fold higher than those in the surrounding endolymph within hours of systemic administration and persists for months owing to high-affinity phospholipid binding (Hailey et al., 2017). This intracellular retention initiates a broadly sequential, though temporally overlapping, cascade of injury: mitochondrial targeting and disruption of oxidative phosphorylation (Schacht et al., 2012; Bottger and Schacht, 2013); reactive oxygen species (ROS) generation through iron–aminoglycoside Fenton chemistry and NADPH oxidase 3 (NOX3)-dependent pathways (Schacht et al., 2012; Sha and Schacht, 1999; Mukherjea et al., 2011); a bidirectional amplification loop between oxidative damage and calcium dysregulation (Esterberg et al., 2013; Tabuchi et al., 2011); and activation of multiple cell death programs, including caspase-mediated apoptosis (Tabuchi et al., 2011; Cunningham et al., 2002), RIPK3/MLKL-dependent necroptosis (Ruhl et al., 2019), and iron-dependent ferroptosis (Zheng et al., 2020; Han et al., 2023). The approximate temporal progression from entry (∼0–6 h) through to execution of cell death programs (∼24–72 h) defines a narrowing but clinically meaningful window for intervention. Each stage is discussed in the subsections that follow.

**TABLE 2:** Integrated pathway of aminoglycoside ototoxicity.

Stage	Timeframe	Key processes	Molecular markers	Intervention window
Entry	∼0–6 h	MET channel permeation; endocytosis; TRP channel entry	Fluorescent AG accumulation; endocytic vesicle formation	MET channel blockers (preclinical); endocytosis inhibitors (preclinical)
Mitochondrial targeting	∼2–12 h	Binding to 12S rRNA; protein synthesis inhibition; initial ROS production	Mitochondrial membrane depolarization/ultrastructural disruption; ↓ ATP production; ↑ superoxide	Alternative ribosome binders (preclinical); mitochondrial protectants (preclinical)
Oxidative amplification	∼6–24 h	Iron–AG complex formation; lipid peroxidation; GSH depletion	4-HNE adducts; protein carbonylation ↑; GSH:GSSG ratio ↓	Antioxidants; iron chelators; GSH precursors
Ion dysregulation	∼12–36 h	↑ intracellular Ca^2+^; altered membrane conductance and ion homeostasis; membrane permeabilization	Ca^2+^ waves; membrane potential changes; phospholipid scrambling	Ca^2+^ chelators; ion channel modulators
Cell death execution	∼24–72 h	Cytochrome c release; caspase activation; necroptosis activation; ferroptosis (iron-dependent lipid peroxidation)	TUNEL-positive nuclei; activated caspase-3; RIPK3/MLKL translocation; lipid peroxidation markers (e.g., 4-HNE)	Anti-apoptotic agents (preclinical); caspase inhibitors (preclinical); RIPK1 inhibitors (preclinical); ferroptosis inhibitors (preclinical)

Timeframes represent approximate, overlapping windows derived primarily from experimental (*in vitro* and animal) models and should not be interpreted as precise clinical kinetics. Values vary by dose, compound, species, and developmental stage. Interventions marked “preclinical” have not been validated in human clinical trials. Molecular markers represent commonly reported mechanistic features rather than universal or sequentially obligatory events. Stages may overlap and vary according to dose, compound, species, and developmental context. Arrows indicate direction of change (↑ increase; ↓ decrease).

Abbreviations: AG, aminoglycoside; ATP, adenosine triphosphate; Ca^2+^, calcium ion; GSH, reduced glutathione; GSSG, oxidized glutathione (glutathione disulfide); GSH: GSSG, ratio of reduced to oxidized glutathione; 4-HNE, 4-hydroxynonenal; MET, mechanoelectrical transduction; MLKL, mixed lineage kinase domain-like protein; RIPK1, receptor-interacting protein kinase 1; RIPK3, receptor-interacting protein kinase 3; ROS, reactive oxygen species; TRP, transient receptor potential; TUNEL, terminal deoxynucleotidyl transferase dUTP nick end labeling.

Aminoglycosides enter cochlear and vestibular hair cells primarily through MET channels located at stereocilia tips. These cation-selective channels, composed of transmembrane channel-like proteins TMC1 and TMC2, normally transduce mechanical stimuli into electrical signals (Pan et al., 2018). The polybasic nature of aminoglycosides facilitates permeation through these channels, with smaller molecules, such as gentamicin, showing higher entry rates than larger compounds, such as amikacin. This differential permeability has clinical implications for drug selection.

Counterintuitively, sound itself promotes drug uptake: when stereocilia deflect, MET channels open more widely, increasing aminoglycoside entry. Functional MET channels are required for aminoglycoside ototoxicity, and increased channel open probability during acoustic stimulation enhances drug entry (Alharazneh et al., 2011). While clinical outcome data are limited, experimental and observational evidence suggest that the ambient noise in NICUs may be enough to worsen drug entry (Zimmerman and Lahav, 2013; Brown, 2009). Additional uptake routes are also relevant. Transient receptor potential (TRP) channels, notably TRPV1 and TRPV4, are upregulated during inflammation, which may explain why concurrent infection seems to heighten the risk of ototoxicity (Cross et al., 2015).

Once inside, aminoglycosides accumulate within hair cells to concentrations that persist for months owing to high-affinity binding to phosphatidylinositol 4,5-bisphosphate (Huth et al., 2011; Hailey et al., 2017). Advanced imaging reveals that aminoglycosides rapidly access the cytosol of hair cells, where a proportion is subsequently sequestered into endosomes and lysosomes; disruption of this lysosomal trafficking potentiates hair cell death (Hailey et al., 2017). This prolonged retention explains why ototoxicity can persist even after treatment stops, a factor clinicians need to consider when deciding how long to monitor patients.

### Mitochondrial dysfunction

Aminoglycosides preferentially localize to mitochondria by binding to the 12S ribosomal RNA, which is encoded by the mitochondrially encoded gene MT-RNR1. This binding disrupts mitochondrial protein synthesis, which is essential for oxidative phosphorylation (Hobbie et al., 2008). The structural similarity between bacterial 16S rRNA and mitochondrial 12S rRNA, reflecting evolutionary origins, underlies both therapeutic efficacy against bacteria and ototoxicity to host cells.

Structural studies using cryo-electron microscopy have mapped the binding sites of aminoglycosides on mitochondrial ribosomal subunits (Bottger and Schacht, 2013). Upon binding, aminoglycosides cause misreading of the genetic code through conformational changes that affect codon-anticodon recognition (Hobbie et al., 2008), premature termination of protein synthesis, and downstream inhibition of electron transport chain complexes I, III, and IV, which contain mitochondrially encoded subunits (Schacht et al., 2012; Rybak and Ramkumar, 2007).

**FIGURE 4:**
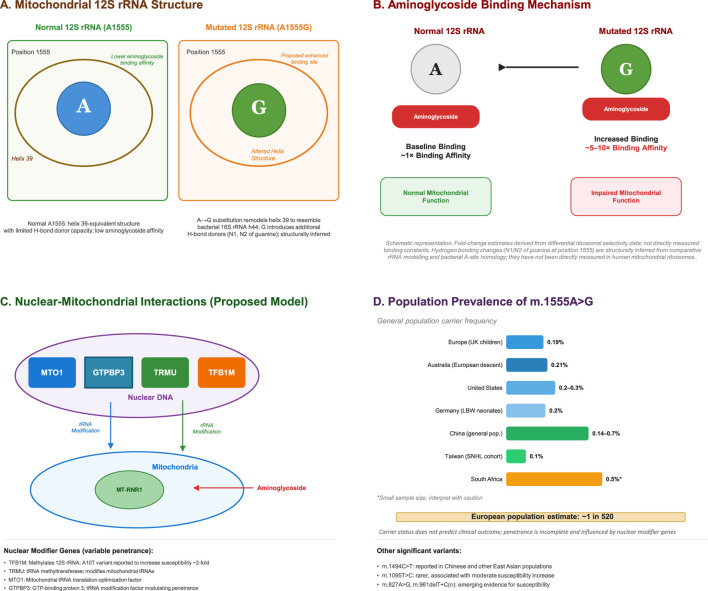
Genetic susceptibility to aminoglycoside ototoxicity: structural basis, nuclear modifiers, and population prevalence of the MT-RNR1 m.1555A>G variant. **(A)** Comparison of normal 12S rRNA (A1555, left) and mutated 12S rRNA (A1555G, right) at position 1,555 within helix 39. In the normal configuration, position 1,555 encodes adenine, producing a helix structure with limited hydrogen-bond donor capacity and low aminoglycoside binding affinity. The A→G substitution remodels helix 39 to structurally resemble the bacterial 16S rRNA decoding site (h44); guanine at position 1,555 introduces additional hydrogen-bond donors (N1 and N2 of guanine), enhancing aminoglycoside affinity (Hobbie et al., 2008; Guan et al., 2000; Guan, 2011). Hydrogen bonding changes are structurally inferred from comparative rRNA modelling and bacterial A-site homology; they have not been directly measured in human mitochondrial ribosomes. **(B)** Functional consequences of the structural change. Normal 12S rRNA (A1555) exhibits baseline aminoglycoside binding (∼1× affinity), preserving mitochondrial protein synthesis and function. Mutated 12S rRNA (A1555G) shows substantially increased aminoglycoside binding (∼5–10× affinity), resulting in impaired mitochondrial function even at therapeutic drug concentrations. Affinity values are schematic representations derived from differential ribosomal selectivity data rather than directly measured binding constants (Hobbie et al., 2008; Guan et al., 2000; Guan, 2011). **(C)** Proposed model of nuclear–mitochondrial interactions modulating penetrance of the m.1555A>G variant. Four nuclear modifier genes influence the phenotypic expression of MT-RNR1-associated ototoxicity: MTO1, GTPBP3, and TRMU participate in mitochondrial tRNA modification pathways; TFB1M methylates 12S rRNA at the mitochondrial ribosome (A10T variant of TFB1M reported to increase susceptibility approximately 2-fold) (Gao et al., 2017; Bykhovskaya et al., 2004). Aminoglycoside binding to the MT-RNR1-encoded 12S rRNA in the presence of these nuclear modifiers contributes to the variable and incomplete penetrance observed among carrier families. Carrier status does not predict clinical outcome; nuclear genetic background, environmental factors, and aminoglycoside exposure together influence penetrance. **(D)** General population carrier frequency of the m.1555A>G variant by population. European and US cohorts report consistent frequencies of approximately 0.19%–0.3%, while Chinese general population studies report a wider range of 0.14%–0.7%. The Taiwan estimate (0.1%) derives from a sensorineural hearing loss (SNHL) cohort rather than a general population screen and should be interpreted accordingly (Wu et al., 2007). The South African estimate (0.5%) is based on a small sample and should be interpreted with caution (Bardien et al., 2009). The European population estimate of approximately 1 in 520 is derived from the United Kingdom ALSPAC birth cohort (Bitner-Glindzicz et al., 2009). Among hearing-impaired populations, carrier frequency is substantially higher, particularly in East Asian cohorts, reflecting ascertainment bias. Other susceptibility variants are shown, including m.1494C>T (reported in East Asian populations), m.1095T>C (rarer, moderate susceptibility increase), and m.827A>G and m.961delT + C(n) (emerging evidence). Data sources: Population prevalence data from Bitner-Glindzicz et al. (2009), Vandebona et al. (2009), Gopel et al. (2014), Lu et al. (2010), Wu et al. (2007), Bardien et al. (2009), and Gaafar et al. (2024); prevalence table adapted from Usami & Nishio (GeneReviews) (Usami and Nishio, 1993). Hearing-impaired cohort frequencies from Guan et al. (2000) and Wu et al. (2007). Structural and binding concepts from Guan (2011), Guan et al. (2000), and Hobbie et al. (2008). Abbreviations: ALSPAC, Avon Longitudinal Study of Parents and Children; GTPBP3, GTP-binding protein 3; LBW, low birth weight; MT-RNR1, mitochondrially encoded 12S ribosomal RNA; MTO1, mitochondrial tRNA translation optimization 1; SNHL, sensorineural hearing loss; TFB1M, transcription factor B1, mitochondrial; TRMU, tRNA 5-methylaminomethyl-2-thiouridylate methyltransferase.

The MT-RNR1 m.1555A>G (Figures 4A,B) variant substantially amplifies this mitochondrial vulnerability, as discussed in the Genetic Susceptibility section below. Aminoglycosides also disrupt mitochondrial dynamics by inhibiting fusion, leading to fragmented mitochondrial networks with compromised function (Esterberg et al., 2013). Live-cell imaging shows mitochondrial fragmentation beginning several hours before detectable hair cell death, suggesting a window for early intervention (Hailey et al., 2017; Esterberg et al., 2013).

### Oxidative stress cascades

Aminoglycosides generate reactive oxygen species through multiple converging mechanisms. Iron-aminoglycoside complexes catalyze Fenton reactions, converting hydrogen peroxide to highly reactive hydroxyl radicals that damage lipids, proteins, and DNA (Sha and Schacht, 1999). Simultaneously, drug binding activates NOX3, which is highly expressed in cochlear tissue, thereby generating superoxide anions (Schacht et al., 2012; Mukherjea et al., 2011).

Cochlear hair cells possess limited intrinsic antioxidant capacity, with lower glutathione (GSH) concentrations and reduced catalase activity relative to many other cell types, and a restricted ability to regenerate reduced GSH from oxidized forms (Schacht et al., 2012; Sha and Schacht, 1999). In neonates, this vulnerability is compounded by developmental immaturity of systemic antioxidant systems, including lower circulating GSH and cysteine concentrations in early postnatal life (Jain et al., 1995). Temporal studies document ROS accumulation preceding morphological changes by 6–12 h (Esterberg et al., 2013). The resulting oxidative damage includes lipid peroxidation, protein carbonylation, and mitochondrial DNA damage. The mitochondrial genome is notably vulnerable due to its proximity to electron transport chain-generated ROS, its lack of protective histones, and its limited repair mechanisms (Schacht et al., 2012).

Calcium dysregulation amplifies oxidative injury through a bidirectional positive feedback loop. Initial mitochondrial ROS production triggers calcium release from intracellular stores, which, in turn, further stimulates mitochondrial ROS production while activating calcium-dependent proteases and phospholipases (Esterberg et al., 2013; Tabuchi et al., 2011). This amplification cascade accelerates cellular damage once initiated, emphasizing the importance of early intervention.

### Cell death pathways

Hair cell death proceeds through multiple interacting mechanisms, with the dominant pathway determined by drug concentration, exposure duration, and cellular stress levels. At lower concentrations or shorter exposures, classical apoptotic pathways predominate, characterized by caspase activation, chromatin condensation, and cell shrinkage without inflammation (Tabuchi et al., 2011). Higher concentrations or prolonged exposure may engage necrotic or necrosis-like features, characterized by cellular swelling, membrane rupture, and inflammatory responses (Forge and Li, 2000).

Intrinsic (mitochondrial) apoptosis is initiated by mitochondrial damage, leading to outer membrane permeabilization and cytochrome c release. Formation of the apoptosome activates executioner caspases 3 and 7. Molecular studies demonstrate early pro-apoptotic signaling, including BAX activation and translocation to mitochondria following aminoglycoside exposure (Cunningham et al., 2002). Extrinsic apoptosis via upregulation of death receptors (Fas/FasL) activates caspase-8 and has been proposed as a contributing pathway, especially in the context of inflammation-potentiated ototoxicity (Tabuchi et al., 2011).

Regulated necrosis pathways, including necroptosis dependent on RIPK1/RIPK3 and mixed lineage kinase domain-like protein (MLKL), may be activated in parallel with or when apoptotic signaling is inhibited (Ruhl et al., 2019). Ferroptosis, an iron-dependent form of cell death characterized by lipid peroxidation, has emerged as another contributing mechanism, given the central role of iron-catalyzed ROS generation in aminoglycoside toxicity; liproxstatin-1 protects both HEI-OC1 cells and cochlear hair cells from neomycin-induced ferroptotic damage (Zheng et al., 2020; Han et al., 2023). In practice, these pathways operate concurrently rather than sequentially, with relative contributions varying by exposure intensity and developmental stage (Ruhl et al., 2019).

The temporal progression follows an approximately predictable pattern amenable to intervention (Table 2): drug entry via MET channels (∼0–6 h), mitochondrial targeting with disruption of oxidative phosphorylation and initial ROS production (∼2–12 h), oxidative amplification featuring iron–aminoglycoside-mediated damage and GSH depletion (∼6–24 h), calcium dysregulation (∼12–36 h), and execution of cell death programs (∼24–72 h) (Huth et al., 2011; Esterberg et al., 2013). These phases overlap substantially, and real-time imaging confirms that death follows a base-to-apex gradient, with basal outer hair cells showing earlier activation of death pathways than apical cells (Forge and Schacht, 2000; Huth et al., 2011).

### Agent-specific toxicity and drug interactions

Different aminoglycosides show distinct toxicity profiles (Table 3). Although the cellular and molecular basis for agent-specific differences in cochlear versus vestibular toxicity is not fully established, differential tissue drug concentrations and uptake rates via MET channels do not appear to explain these patterns (Forge and Schacht, 2000; Alharazneh et al., 2011). Differences in intracellular binding affinity, subcellular distribution, and relative potency of ROS generation among aminoglycoside compounds have been proposed as contributing factors, but definitive mechanistic studies comparing agents under equivalent exposure conditions are lacking. These categories represent relative trends rather than quantitative effect sizes. Gentamicin preferentially damages vestibular type I hair cells, causing balance disturbances with relative cochlear sparing at therapeutic doses (Forge and Schacht, 2000). Amikacin is classically considered more cochleotoxic than gentamicin, consistent with differences in molecular structure among aminoglycoside compounds (Forge and Schacht, 2000; Selimoglu, 2007). Neomycin generally produces more rapid hair cell injury in experimental hair-cell preparations than gentamicin at comparable concentrations, consistent with neomycin’s greater intrinsic ototoxic potency (Forge and Schacht, 2000; Huth et al., 2011). Fluorescent gentamicin studies demonstrate trafficking of systemically administered drug into the cochlea and hair cells (Wang and Steyger, 2009).

**TABLE 3:** Relative cochlear and vestibular toxicity patterns of aminoglycosides.

Agent	Cochleotoxicity	Vestibulotoxicity	Onset speed	Primary pattern
Gentamicin	++	++++	Moderate	Preferential vestibular injury reported; cellular uptake and retention patterns differ by tissue
Streptomycin	+	++++	Slow	Preferential vestibular accumulation is described in experimental models
Amikacin	+++	+	Moderate	Higher cochlear injury potential in experimental models; outer hair cell uptake implicated
Kanamycin	++++	+	Rapid	Enhanced cochlear penetration is described; ROS and oxidative stress pathways are implicated
Neomycin	++++	++	Very rapid	Rapid ROS generation in experimental models; mitochondrial pathways implicated
Tobramycin	+++	++	Moderate	Relatively balanced cochlear and vestibular distribution reported

+ = minimal effect; ++ = mild; +++ = moderate; ++++ = severe. Plus symbols indicate relative patterns of cochleotoxicity and vestibulotoxicity synthesized from patterns consistently reported across experimental hair-cell studies and clinical observational literature, rather than representing quantitative comparative effect sizes. “Onset speed” reflects the relative time to detectable functional or histologic injury under typical experimental or clinical exposure conditions and does not represent a fixed temporal threshold. Patterns may vary depending on dose, cumulative exposure, route of administration (e.g., intravenous vs. inhaled), population, and monitoring methodology.

Exposure metrics: Comparative toxicity patterns across agents are derived from studies using heterogeneous exposure metrics (Cmax, trough concentration, AUC, cumulative dose, and/or treatment duration). Rankings should be interpreted in the context of this methodological variability.

Abbreviations: ROS, reactive oxygen species.

The co-administration of other ototoxic medications significantly increases the risk. Specific risk medications are discussed in detail in the Drug-Related Factors section below.

## Risk factors and high-risk populations

### Genetic susceptibility

The MT-RNR1 m.1555A>G variant represents the most clinically actionable risk factor for aminoglycoside ototoxicity (Figures 4A–C). This mitochondrial DNA variant alters 12S rRNA structure to more closely resemble bacterial 16S rRNA, enhancing aminoglycoside binding approximately 5- to 10-fold at the ribosomal decoding site (McDermott et al., 2022b; Hobbie et al., 2008; Rahman et al., 2012; Guan et al., 2000). The m.1555A>G variant is most commonly homoplasmic, consistent with its maternal mitochondrial inheritance, though variable levels of heteroplasmy have been documented within and across carrier families (del Castillo et al., 2003). Preliminary data suggest a correlation between mutation load and phenotypic severity, with mutation burdens below approximately 20% of mutant copies associated with absent or mild hearing loss, whereas all subjects with mutation loads above 52% had hearing loss (del Castillo et al., 2003); however, this correlation was not consistent across all families studied, and peripheral blood heteroplasmy levels may not accurately reflect mutation load in the inner ear. Current guidance recommends that all identified carriers avoid aminoglycosides regardless of heteroplasmy level (McDermott et al., 2022b). Carriers exhibit a very high likelihood of sensorineural hearing loss following aminoglycoside exposure, even at therapeutic serum concentrations (Rahman et al., 2012; Prezant et al., 1993). Even brief exposure, including single-dose administration, has been reported to precipitate profound, irreversible bilateral sensorineural hearing loss in carriers (Vandebona et al., 2009). This risk is not predicted by standard therapeutic drug monitoring (McDermott et al., 2022b). Therefore, routine pharmacokinetic monitoring provides limited clinical utility for these individuals. For identified MT-RNR1 carriers, aminoglycoside avoidance rather than pharmacokinetic optimization is the primary risk mitigation strategy. In contrast, for the much larger non-carrier population, TDM retains its established role in minimizing cumulative exposure and optimizing dosing, as discussed in the Therapeutic Drug Monitoring section below.

Population prevalence varies by ethnicity (Figure 4D): approximately 0.19% (1 in 520) in a European pediatric cohort (Bitner-Glindzicz et al., 2009) and 0.26% (1 in 385) in a British adult cohort (Rahman et al., 2012). General population prevalence in European and Australian cohorts ranges from approximately 0.17%–0.26% (Rahman et al., 2012; Vandebona et al., 2009; Bitner-Glindzicz et al., 2009; Gopel et al., 2014). However, robust data from non-European general populations remain limited. In NICU settings where most infants receive aminoglycosides, the population-level impact is substantial: in the United Kingdom, an estimated 180 neonates per year are at risk of aminoglycoside-induced hearing loss due to undetected m.1555A>G carriage (McDermott et al., 2022a), with proportionally larger numbers expected in countries with higher birth rates.

Additional mitochondrial variants confer variable susceptibility. The m.1494C>T mutation causes structural changes analogous to those of m.1555A>G at the A-site of 12S rRNA, though the reported penetrance of hearing loss following aminoglycoside exposure appears lower and more variable. Four nuclear modifier genes: MTO1, GTPBP3, TRMU, and TFB1M have been identified, and the variable penetrance of hearing loss observed within and among families carrying m.1555A>G suggests that nuclear genetic background modulates clinical expression, though precise effect sizes for individual modifiers have not been robustly quantified in adequately powered studies. Aminoglycosides enter cochlear hair cells primarily through MET channels rather than classical drug transporters (Huth et al., 2011); accordingly, pharmacogenetic variation in transporter genes is unlikely to modulate ototoxic susceptibility. Variants in oxidative stress response genes such as GSTP1 and SOD2 have been proposed as potential susceptibility modifiers, but studies of glutathione S-transferase null genotypes (GSTM1, GSTT1) found no influence on aminoglycoside ototoxicity (Palodetto et al., 2010; Gaafar et al., 2024). Genetic association studies examining human variants in other mechanistic pathway genes, including those encoding MET channel components (TMC1, TMC2), NOX3, and regulated cell death mediators (RIPK3, MLKL), have not been reported, representing a gap in the pharmacogenomic literature (Pan et al., 2018; Alharazneh et al., 2011; Mukherjea et al., 2011; Ruhl et al., 2019). Taken together, these candidate gene associations require validation in adequately powered pediatric cohorts before clinical conclusions can be drawn.

### Age-related vulnerabilities

Pediatric populations show age-related differences in ototoxicity susceptibility, with neonates, particularly preterm neonates, facing the highest risk. The incidence of sensorineural hearing loss in very low birthweight infants has been reported to range from approximately 0.4%–5% (Diepstraten et al., 2021; Zimmerman and Lahav, 2013), substantially higher than in the general pediatric population. However, the independent contribution of aminoglycoside exposure versus other NICU-associated risk factors remains difficult to disentangle. Low gestational age, low birthweight, hyperbilirubinemia, asphyxia, cumulative ototoxic drug exposure, and duration of aminoglycoside treatment interact to increase hearing loss risk, with younger and more premature neonates accumulating more risk factors over longer treatment courses (Diepstraten et al., 2021; Zimmerman and Lahav, 2013). Susceptibility decreases with postnatal maturation but remains clinically relevant through early childhood.

Several physiological factors combine to explain why neonates are so vulnerable. Renal immaturity significantly affects aminoglycoside clearance. Glomerular filtration rate (GFR) in preterm neonates is markedly reduced, with the most premature infants (born at ∼26 weeks’ gestational age) showing values several-fold lower than those of term neonates at 1 month of age; GFR matures rapidly but variably over the first weeks to months of life, approaching adult values by one to 2 years of age (De Cock et al., 2012; Wu et al., 2024; Iacobelli and Guignard, 2021). Higher total body water in very preterm neonates increases volume of distribution relative to adults; gentamicin volume of distribution ranges from 0.5 to 0.7 L/kg in preterm neonates compared with approximately 0.25 L/kg in young adults (Fuchs et al., 2014; Rivera-Chaparro et al., 2017). Developmental immaturity of the blood-labyrinth barrier, including incomplete tight junction formation in strial capillaries, increases cochlear permeability in immature animals compared with adults (Suzuki et al., 1998; Shi, 2016), although the relative contribution of barrier permeability versus MET channel entry to aminoglycoside cochlear uptake in neonates remains incompletely characterized (Alharazneh et al., 2011). Neonatal antioxidant defenses are reduced compared with those of adults, with GSH levels in preterm infants further compromised (Jain et al., 1995).

Infants aged 1–12 months remain at elevated risk relative to older children. Vestibular dysfunction during this developmental window is of particular concern because the vestibular system plays a critical role in the emergence of postural control and gross motor milestones; vestibular hypofunction in early infancy has been associated with progressive delay in motor development (Wiener-Vacher et al., 2018; Wiener-Vacher et al., 2013; Rine et al., 2004). Children aged 1–5 years generally require higher weight-adjusted aminoglycoside doses than older children and adolescents to achieve equivalent peak serum concentrations, reflecting their proportionally larger volume of distribution and higher weight-normalized clearance. Despite these pharmacokinetic differences, clinical ototoxicity remains relevant in early childhood, suggesting that developmental factors beyond drug disposition contribute to susceptibility.

### Drug-related factors

Cumulative aminoglycoside exposure is one of the strongest predictors of ototoxicity. Both treatment duration and total dose correlate with hearing loss incidence, with prolonged courses markedly increasing the risk (Beaubien et al., 1989; Modongo et al., 2015; O'Sullivan et al., 2017); cumulative AUC is a stronger predictor than peak or trough concentrations alone, as discussed in the Therapeutic Drug Monitoring section below. Repeated treatment courses compound risk even when individual courses remain within recommended parameters, a finding of particular concern in populations requiring recurrent aminoglycoside therapy, such as patients with cystic fibrosis (Garinis et al., 2017a). Careful documentation of cumulative and lifetime aminoglycoside exposure is therefore important for risk assessment.

Concurrent ototoxic medications substantially increase risk (Table 4). Loop diuretics are well-established synergists; co-administration with aminoglycosides enhances cochleotoxicity, likely through loop diuretic-mediated disruption of the stria vascularis that increases blood-labyrinth barrier permeability to aminoglycosides (Shi, 2016; Rybak, 1993; Bates et al., 2002). The combination of loop diuretics and aminoglycosides is associated with greater ototoxicity than aminoglycosides alone, although the magnitude of increased risk varies across studies (Fuchs et al., 2014; Rivera-Chaparro et al., 2017). Loop diuretics, particularly furosemide, are frequently co-administered with aminoglycosides in critically ill neonates not as a deliberate combination, but because these patients often require diuretic therapy for fluid overload, bronchopulmonary dysplasia, or hemodynamically significant patent ductus arteriosus concurrent with aminoglycoside treatment for suspected or confirmed sepsis (Cantey et al., 2015). Furosemide is the best-characterized synergist, acting by reducing endocochlear potential and disrupting blood-labyrinth barrier integrity, thereby enhancing aminoglycoside access to hair cells (Rybak, 1993; Bates et al., 2002).

**TABLE 4:** Synergistic interactions with aminoglycosides.

Factor	Mechanism of synergy	Reported risk pattern†	Exposure timing
Loop diuretics	Blood–labyrinth barrier disruption; altered endolymphatic potential; reduced cochlear blood flow	High: substantially increased clinical risk reported; cochlear drug concentrations elevated 2–3-fold in animal models	Concurrent or near-concurrent administration
Vancomycin	Additive mitochondrial toxicity; competitive renal elimination; enhanced oxidative stress	Low–Moderate: association reported in neonatal cohorts; confounded by illness severity and indication; outcome often screening referral rather than confirmed permanent hearing loss	Concurrent administration
Platinum compounds	Complementary ROS generation; DNA damage amplification; shared uptake mechanisms	High: substantially increased risk reported across clinical and experimental studies	Prior or concurrent administration
Inflammation	TRP channel upregulation; potentiated ROS production; pro-inflammatory cytokine release	Moderate–High: increased risk reported; magnitude varies by cohort and outcome definition	Preceding inflammatory states or concurrent systemic illness
Noise exposure	Increased MET channel open probability; additional ROS generation; reduced cochlear blood flow	Moderate: additive injury reported in experimental models	Concurrent or peri-exposure
Hypoxia/Ischemia	ATP depletion; impaired protective responses; enhanced iron release	Moderate: additive injury reported in experimental models	Before or concurrent with aminoglycoside exposure

† Reported risk pattern levels (Low, Moderate, High) represent approximate patterns derived from observational and experimental literature (Schacht et al., 2012; Cross et al., 2015; Rybak and Ramkumar, 2007; Rybak, 1993; Bates et al., 2002; Brummett et al., 1990; Vella-Brincat et al., 2011; Li and Steyger, 2009; Koo et al., 2015). Estimates vary by outcome definition (e.g., screening referral vs. audiometry-confirmed sensorineural hearing loss), follow-up duration, and confounding by illness severity. Values should not be interpreted as precise quantitative estimates.

‡ Exposure metrics: Risk patterns are derived from studies using variable exposure metrics and outcome definitions. Where specific studies are referenced, the exposure metric used (e.g., Cmax, trough, AUC, cumulative dose) is described in the corresponding manuscript text.

Abbreviations: AG, aminoglycoside; ATP, adenosine triphosphate; MET, mechanoelectrical transduction; ROS, reactive oxygen species.

Vancomycin, commonly co-administered in neonatal sepsis protocols, augments aminoglycoside ototoxicity in preclinical models (Brummett et al., 1990). Clinical data from neonatal cohorts suggest an association between vancomycin exposure and increased hearing screening failure rates, though the evidence remains mixed and confounded by illness severity (Vella-Brincat et al., 2011; Lestner et al., 2016; Marissen et al., 2020). Cisplatin and high-dose salicylates are well-recognized ototoxins, and macrolide antibiotics have also been associated with hearing changes; potential additive risk in polypharmacy settings warrants vigilance (Rybak and Ramkumar, 2007).

Renal function is critical because it determines drug exposure. Aminoglycosides are eliminated almost entirely by glomerular filtration; accordingly, renal function is the principal determinant of systemic drug concentrations and, consequently, inner-ear drug accumulation (Turnidge, 2003). Acute kidney injury creates a positive feedback cycle in which impaired clearance elevates drug concentrations, which in turn exacerbate nephrotoxicity (Rybak, 1993). In neonates, the rapid maturation of GFR during the first weeks of life necessitates frequent monitoring and dose-interval adjustment (De Cock et al., 2012).

Environmental factors, including NICU ambient noise levels, may potentiate ototoxicity through the activity-dependent MET channel uptake mechanism described in the Cellular Entry section above; ambient sound levels in NICUs typically exceed recommended limits by several-fold, and preclinical evidence demonstrates that sound exposure enhances aminoglycoside cochlear uptake in a synergistic rather than additive manner (Li and Steyger, 2009; Garinis et al., 2017b). Concurrent illness severity independently affects risk; sepsis, respiratory failure, and hyperbilirubinemia have been associated with increased hearing loss in aminoglycoside-treated neonates, potentially through inflammation-mediated enhancement of cochlear drug uptake (Koo et al., 2015; Jiang et al., 2017).

## Prevention and monitoring strategies

### Genetic screening

Rapid point-of-care MT-RNR1 screening has been available since the CE marking of the Genedrive MT-RNR1 ID Kit in 2019, with clinical implementation in United Kingdom NICU settings following the PALOH trial (McDermott et al., 2022a) and subsequent NICE Early Value Assessment recommendation in 2023. Pre-treatment identification of MT-RNR1 mutation carriers offers the most direct and targeted prevention strategy for genetically susceptible individuals. Point-of-care testing platforms such as the Genedrive MT-RNR1 ID Kit deliver results within approximately 26–30 min from a buccal swab, enabling real-time prescribing decisions even in urgent scenarios (McDermott et al., 2022a). In the PALOH trial, 80.6% of neonates prescribed antibiotics were successfully genotyped before or concurrently with antimicrobial initiation, with no significant delay in time-to-antibiotic administration (McDermott et al., 2022a). Of the 424 neonates who were eligible for antibiotic therapy and were successfully genotyped, 8 (1.89%) received a variant (VAR) result on the Genedrive POCT. Sanger sequencing confirmation identified 5 of these as false positives, subsequently attributed to incomplete cartridge insertion causing light ingress during the melt phase, a defect resolved by a subsequent cartridge redesign (McDermott et al., 2022a). The remaining 3 (0.71% of those genotyped) were confirmed as true MT-RNR1 m.1555A>G variant carriers; all three avoided aminoglycoside antibiotics and received alternative cephalosporin-based regimens (McDermott et al., 2022a). These false-positive results yielded a real-world analytical specificity of 99.2% (95% CI, 98.0%–99.7%), compared with the 100% specificity observed in preclinical validation. The authors note that, given the worldwide use of aminoglycosides in more than 7 million neonates annually, widespread adoption of MT-RNR1 point-of-care testing could potentially avoid thousands of cases of aminoglycoside-induced ototoxicity each year (McDermott et al., 2022a). Several United Kingdom National Health Service trusts have since implemented universal NICU protocols, and a NICE Early Value Assessment concluded that the test has the potential to be clinically effective and cost-effective. However, the recommendation remains conditional on further evidence generation (NICE, 2023).

The Clinical Pharmacogenetics Implementation Consortium (CPIC) guideline recommends that carriers of the m.1555A>G variant avoid aminoglycosides whenever possible (McDermott et al., 2022b). Alternative antibiotics for gram-negative coverage include extended-spectrum cephalosporins (ceftazidime, cefepime) and carbapenems (meropenem); fluoroquinolones may be considered in older children and adults, but are generally avoided in neonates due to concerns regarding cartilage toxicity (McDermott et al., 2022b). In rare life-threatening infections without viable alternatives, aminoglycoside exposure may still be unavoidable, necessitating intensive mitigation. In such circumstances, the CPIC guideline recommends administration for the shortest possible duration, under expert supervision, with therapeutic drug monitoring and serial audiological assessment, recognizing that ototoxic risk remains substantial even with these precautions (McDermott et al., 2022b).

Practical barriers include test availability, workflow integration, staff training, uncertainty about alternative antibiotic protocols, and concerns about the familial implications of identifying a maternally inherited mitochondrial variant, which may require genetic counseling (Rigobello et al., 2023). Early cost-effectiveness analyses suggest favorable outcomes when screening costs are weighed against lifetime hearing-related healthcare expenditures, though full economic evaluations remain limited (NICE, 2023). Where resources preclude universal screening, a risk-stratified approach, screening patients with a family history of aminoglycoside-related hearing loss or those facing prolonged or repeated treatment, may represent a pragmatic intermediate step (McDermott et al., 2022b).

### Therapeutic drug monitoring

Traditional therapeutic drug monitoring (TDM) for aminoglycosides focuses on peak and trough concentration measurements. Peak concentrations, typically measured 30 min after completion of the intravenous infusion, serve as surrogates for concentration-dependent bactericidal activity, as reflected in the Cmax/MIC ratio; targets of ≥8–10 are considered optimal for clinical efficacy (Moore et al., 1987). Target ranges are agent- and regimen-specific; for example, gentamicin peak targets are 5–10 μg/mL with conventional multiple daily dosing and ≥10–12 μg/mL with extended-interval protocols, with corresponding targets for tobramycin and amikacin scaled accordingly (Turnidge, 2003; Pinder et al., 2002). Although gentamicin is the most frequently studied aminoglycoside in neonatal TDM, the same pharmacokinetic principles apply across the class: concentration-dependent killing, predominantly renal elimination, and exposure-driven toxicity. Accordingly, TDM frameworks are routinely adapted for tobramycin and amikacin using agent-specific targets and dosing intervals. Trough concentrations are monitored primarily to minimize drug accumulation and nephrotoxicity risk, with targets of <2 μg/mL for conventional dosing and <1 μg/mL for extended-interval regimens for gentamicin and tobramycin, and <10 μg/mL for amikacin (Turnidge, 2003; Pinder et al., 2002). Modongo et al. (2015) demonstrated that when peak and trough concentrations were forced as primary predictors of ototoxicity, discriminative performance was no better than chance (AUROC 0.46); similarly, Setiabudy et al. found no relationship between aminoglycoside trough concentrations and ototoxicity in neonates (Setiabudy et al., 2013). In contrast, cumulative AUC and treatment duration were the dominant predictors. This has important implications for TDM strategy, as conventional peak/trough monitoring may provide reassurance regarding nephrotoxicity avoidance and efficacy optimization while offering limited predictive value for ototoxicity risk (Roger et al., 2021).

These findings have prompted interest in AUC-guided monitoring approaches. For aminoglycoside efficacy, the Cmax/MIC ratio remains the primary pharmacodynamic driver; however, for toxicity prediction, cumulative drug exposure, particularly cumulative AUC over the treatment course, appears more informative than isolated peak or trough measurements (Modongo et al., 2015), a finding further supported by the AMINO III international critical care survey (Roger et al., 2021). Cumulative exposure serves as a toxicity-relevant metric across the class, supported by evidence from amikacin MDR-TB cohorts and populations receiving repeated aminoglycoside courses, such as cystic fibrosis. This is particularly relevant for amikacin, where prolonged treatment courses in drug-resistant tuberculosis make cumulative exposure tracking essential. Limited-sampling strategies using two concentration measurements, typically one early post-infusion sample and one drawn 6–12 h post-dose, have been proposed to estimate individual AUC values without requiring intensive pharmacokinetic sampling, though prospective validation in neonatal populations remains limited (Holford and Buclin, 2012).

Bayesian forecasting represents a further refinement, combining population pharmacokinetic models with individual patient covariates and measured drug concentrations to generate patient-specific pharmacokinetic parameter estimates and optimize dosing. In a retrospective simulation study comparing a Bayesian clinical decision support system (CDSS) with standard-of-care nomogram-based dosing for gentamicin in neonates, Yu et al. (2020), reported that the CDSS maintained peak target attainment above 90% through both initial and adjusted dosing. In contrast, standard-of-care peak target attainment fell from 87% at initial dosing to 66% following clinician-directed adjustments. Although the strongest evidence base for Bayesian aminoglycoside dosing in neonates derives from gentamicin studies, validated population pharmacokinetic models and model-based dosing guidelines exist for both gentamicin and tobramycin, and external validation in critically ill neonates confirms applicability across these agents (Hartman et al., 2020). Several commercial model-informed precision dosing (MIPD) platforms now incorporate validated population pharmacokinetic models for gentamicin, tobramycin, and amikacin, facilitating class-wide application of individualized dosing. More broadly, the same model-informed framework can be applied to any aminoglycoside, with concentration targets and sampling timing tailored to the specific agent and dosing regimen. These findings suggest that MIPD tools may improve target attainment, particularly for dose adjustments. However, it should be noted that the Yu et al. (2020) comparison was an *in silico* study rather than a prospective clinical trial. Clinical outcome data directly linking MIPD-guided aminoglycoside dosing to reduced ototoxicity rates remain lacking and represent an important evidence gap (Keizer et al., 2018).

Physiologically based pharmacokinetic (PBPK) modeling offers a complementary approach by integrating developmental physiology, including age-dependent changes in body composition, renal maturation, and drug distribution, with drug-specific physicochemical parameters to predict drug disposition across pediatric age groups (Edginton et al., 2006; Maharaj and Edginton, 2014). While PBPK modeling has been extensively applied to pediatric drug development broadly, aminoglycoside-specific PBPK models are still emerging; most aminoglycoside dose optimization in neonates relies on population pharmacokinetic modeling with Bayesian estimation (Zazo et al., 2022; D'Agate et al., 2021). Future integration of PBPK models incorporating cochlear distribution and blood-labyrinth barrier ontogeny may improve mechanistic prediction of ototoxic risk. Regardless of the modeling framework, standardized institutional protocols that incorporate pharmacokinetic-guided dosing, regular audiological monitoring, and defined escalation pathways are essential for translating these advances into consistent clinical practice. Pharmacist-led TDM services and electronic CDSS tools may facilitate adherence to protocols and reduce dosing variability. However, rigorous evaluation of their impact on clinical outcomes, including ototoxicity rates, is needed (Roger et al., 2021).

### Extended-interval dosing

Extended-interval (once-daily) dosing is widely adopted and preferred in many pediatric settings. The pharmacological rationale rests on the concentration-dependent bactericidal activity of aminoglycosides, whereby higher peak-to-MIC ratios enhance killing efficacy, combined with the saturable nature of aminoglycoside uptake into renal tubular and cochlear hair cells: prolonged drug-free intervals between doses may reduce cumulative intracellular drug loading and thereby the potential for organ toxicity (Contopoulos-Ioannidis et al., 2004; Nestaas et al., 2005).

Meta-analytic evidence consistently supports extended-interval dosing as at least equivalent to multiple daily dosing in efficacy and safety. In the most comprehensive pediatric meta-analysis, Contopoulos-Ioannidis et al., (Contopoulos-Ioannidis et al., 2004), found no significant difference in clinical failure between regimens and noted that secondary nephrotoxicity outcomes significantly favored extended-interval dosing (RR 0.33, 95% CI 0.12–0.89). Regarding ototoxicity, a non-significant trend favoring extended-interval dosing was observed (RR 0.67, 95% CI 0.42–1.07). However, the authors noted that ototoxicity reporting was incomplete across the included trials and that event rates in multiple-daily-dosing arms were very low, limiting statistical power. Meta-analyses in predominantly adult populations have similarly found no significant difference in ototoxicity rates between regimens, while confirming reduced nephrotoxicity risk with extended-interval dosing (Barza et al., 1996). A neonatal-specific meta-analysis by Nestaas et al. (2005) encompassing 16 trials and 823 neonates, confirmed that extended-interval dosing significantly reduced the risk of serum drug concentrations falling outside the therapeutic range, with no significant differences in nephrotoxicity or ototoxicity. Although a clinically meaningful reduction in ototoxicity is biologically plausible given the saturable nature of cochlear uptake, event rates across all three analyses were too low, and audiometric monitoring was too inconsistent to demonstrate this statistically.

Age-specific extended-interval protocols account for developmental pharmacokinetic variability, particularly the maturational changes in GFR and volume of distribution that influence aminoglycoside clearance across gestational and postnatal age. Although much of the neonatal evidence base derives from gentamicin studies, the same principles apply to tobramycin (with closely similar pharmacokinetics) and amikacin, and dosing frameworks are routinely adapted across the class using agent-specific peak targets and dosing intervals. In practice, preterm neonates receive longer intervals (often 36–48 h) than term neonates (often 24–36 h), with interval selection guided by gestational and postnatal age and supported by validated individualization tables that use a single post-dose concentration to determine the interval (Dersch-Mills et al., 2012). A Cochrane systematic review of randomized trials in term neonates confirmed comparable efficacy and significantly lower trough concentrations with extended-interval compared with twice-daily dosing (Rao et al., 2016). In infants and older children, once-daily dosing is commonly used. In contrast, amikacin regimens use proportionally higher peak targets and interval adjustment by age and renal function to reflect its different potency-to-MIC relationship. Across agents, dosing is paired with TDM using agent-specific peak targets (higher for amikacin) and low trough goals to minimize accumulation, as discussed in the preceding section. Beyond pharmacokinetic advantages, extended-interval dosing simplifies administration and can reduce monitoring burden and costs, supporting its broad adoption in pediatric care (Contopoulos-Ioannidis et al., 2004).

### Audiological monitoring

Serial audiological assessment enables the detection of subclinical cochlear injury before it progresses to permanent threshold shifts. As cumulative exposure and therapy duration are key determinants of ototoxic risk, monitoring strategies should be individualized based on anticipated exposure burden and the presence of high-risk features, including prolonged or repeated courses, renal dysfunction, MT-RNR1 carriage, or concurrent ototoxins. Tables 5, 6 summarize recommended auditory and vestibular monitoring approaches by patient age; age groupings are pragmatic, based on testing feasibility, and do not strictly follow ICH E11 (R1) (International Council for Harmonisation of Technical Requirements for Pharmaceuticals for Human Use ICH, 2017) categories used elsewhere in this manuscript. These recommendations are derived from established ototoxicity monitoring frameworks, as formal pediatric aminoglycoside-specific guidelines remain heterogeneous (American Academy of Audiology, 2009).

**TABLE 5:** Age-appropriate auditory monitoring for aminoglycoside ototoxicity.

Age group	Primary assessment	Secondary assessment	Practical considerations	Monitoring frequency
Neonates (0–27 days)	ABR with frequency-specific tone burst stimuli (500–4,000 Hz); prioritize the highest feasible frequencies for early detection of basal cochlear changes	DPOAE with high-frequency emphasis (4–8 kHz)	Testing during natural sleep Portable equipment for bedside assessment Middle ear status verification	Baseline before therapy, when feasible Intensify for prolonged courses (>7–10** **days), repeated courses, renal dysfunction, MT-RNR1 risk, or concurrent ototoxins Post-treatment follow-up for any detected change
Infants (28 days–12 months)	DPOAE (2–8 kHz); ABR for baseline	VRA (6–12 months); behavioral observation (supplementary only)	Testing during optimal alertness Parental assistance for VRA conditioning Objective measures when behavioral is unreliable	Baseline before therapy, when feasible Intensify for prolonged courses, repeated courses, renal dysfunction, MT-RNR1 risk, or concurrent ototoxins Post-treatment follow-up for high-risk or any detected change
Young children (1–5 years)	Conditioned play audiometry; DPOAE (2–8 kHz)	Immittance measures: ABR if behavioral testing is unreliable	Game-based conditioning approaches Brief session duration (15–20 min) Visual reinforcement	Baseline before therapy Intensify for prolonged or repeated courses, renal dysfunction, genetic risk, or concurrent ototoxins Post-treatment follow-up
Older children (≥5 years)	Conventional audiometry with extended high frequencies (where available)	Speech audiometry; DPOAE	Self-report of symptoms Extended high frequencies are most sensitive Functional assessment	Baseline before therapy Intensify for prolonged or repeated courses, renal dysfunction, genetic risk, or concurrent ototoxins Post-treatment follow-up

Age groupings are pragmatic, based on audiology and vestibular testing feasibility, and do not strictly follow ICH E11 (R1) categories used elsewhere in the manuscript. Audiological testing feasibility, rather than pharmacological classification, determines the monitoring strategy and, therefore, the age groupings used here. Monitoring frequency should be individualized based on cumulative exposure, course duration, renal function, genetic susceptibility, and concurrent ototoxic exposures rather than applied as fixed intervals.

Abbreviations: ABR, auditory brainstem response; DPOAE, distortion product otoacoustic emission; ICH, international council for harmonisation; MT-RNR1, mitochondrially encoded 12S ribosomal RNA; VRA, visual reinforcement audiometry.

**TABLE 6:** Age-appropriate vestibular assessment for aminoglycoside monitoring.

Age group	Primary assessment	Secondary assessment	Practical considerations	Indications for testing
Neonates (0–27 days)	Developmental vestibular assessment Positioning reflex testing	Modified cVEMPs, adapted vHIT (specialist center/where available)	Brief assessment during periods of alertness Parental assistance for positioning Specialized infant equipment Developmental milestone surveillance as a universal default	Extended therapy with gentamicin/streptomycin Abnormal motor patterns Known genetic risk (e.g., m.1555A>G carrier)
Infants (28 days–12 months)	Rotational chair with infant visual targets Developmental milestone assessment	Modified VEMPs, positional testing (specialist center/where available)	Testing during optimal alertness Age-appropriate stimuli Brief assessment duration	Extended aminoglycoside exposure Delayed motor milestones Abnormal head/eye movement patterns
Young children (1–5 years)	Modified CTSIB Functional gait assessment	Rotational chair testing; adapted computerized posturography	Play-based assessment approaches Dynamic balance challenges Short testing intervals	Symptoms of imbalance Motor skill regression History of vestibulotoxic aminoglycosides
Older children (≥5 years)	Video head impulse test VEMPs (cervical and ocular)	Computerized dynamic posturography; rotary chair testing	Self-report of symptoms Standard testing protocols Quantitative measurement	Patient-reported dizziness Balance difficulties Extended aminoglycoside therapy

Age groupings are pragmatic, based on audiology and vestibular testing feasibility, and do not strictly follow ICH E11 (R1) categories used elsewhere in the manuscript. Audiological testing feasibility, rather than pharmacological classification, determines the monitoring strategy and, therefore, the age groupings used here. Neonatal and infant vestibular assessment beyond developmental milestone surveillance typically requires access to a specialist center. Specialized tests (e.g., cVEMP, vHIT) may require dedicated equipment and trained personnel. Cumulative exposure, symptomatology, genetic risk factors, developmental stage, and availability of specialized equipment should guide testing indications.

Abbreviations: CTSIB, clinical test of sensory integration for balance; cVEMP, cervical vestibular evoked myogenic potential; MT-RNR1, mitochondrially encoded 12S ribosomal RNA; VEMP, vestibular evoked myogenic potential; vHIT, video head impulse test.

Conventional pure-tone audiometry assesses thresholds at 250–8,000 Hz. Extended high-frequency audiometry (9–20 kHz) may detect early basal cochlear damage before standard frequencies are affected, providing a critical window for intervention (Fausti et al., 1992). Otoacoustic emissions (OAEs) objectively assess outer hair cell function without patient cooperation, making them especially useful in neonates. OAE amplitude reduction can precede detectable threshold shifts on pure-tone audiometry, providing an earlier indicator of outer hair cell injury (Stavroulaki et al., 2002). Auditory brainstem response (ABR) testing evaluates the entire auditory pathway and serves as the gold standard for neonatal hearing assessment in universal hearing screening programs (American Academy of Audiology, 2009). In neonates, automated ABR and OAEs are most practical for serial monitoring, as behavioral audiometry is not feasible in this age group.

Historically proposed schedules include baseline assessment, weekly monitoring during treatment, and follow-up at 1, 3, and 6 months post-treatment to detect delayed-onset or progressive losses (ASHA, 1994), though implementation varies widely in pediatric practice. Although these foundational society guidelines remain widely referenced, more recent population-specific consensus documents have emerged: the International Ototoxicity Management Working Group has published recommendations for aminoglycoside-exposed cystic fibrosis patients that operationalize extended high-frequency audiometry, OAEs, and structured baseline-to-annual follow-up within routine clinical care (Garinis et al., 2021), and the International Society of Paediatric Oncology has issued age-stratified monitoring recommendations for children receiving ototoxic therapies more broadly (Meijer et al., 2021). These reinforce the core monitoring architecture of pre-treatment baseline, on-therapy surveillance, and post-treatment follow-up, while providing more contemporary, operationally detailed frameworks than the earlier guidelines. Despite these recommendations, structured ototoxicity monitoring in pediatric settings remains inconsistently implemented, particularly outside oncology programs (American Academy of Audiology, 2009).

Vestibular function assessment is frequently overlooked, despite well-recognized vestibulotoxicity risk posed by aminoglycosides as a class. Gentamicin and streptomycin are relatively more vestibulotoxic, while tobramycin affects both cochlear and vestibular structures (Leis et al., 2015). In early childhood, undetected vestibular dysfunction may contribute to delayed acquisition of gross motor milestones and postural instability. Video head impulse testing (vHIT) evaluates semicircular canal function; vestibular evoked myogenic potentials (VEMPs) assess otolith organs (Rosengren et al., 2019). Parent-reported balance difficulties and motor delays warrant formal vestibular evaluation in aminoglycoside-exposed children (Rine and Wiener-Vacher, 2013).

### Otoprotective agents

Several compounds have demonstrated otoprotective potential in preclinical models, though clinical translation remains limited, and none are currently recommended as standard of care outside clinical trials. N-acetylcysteine (NAC), a glutathione precursor that augments intracellular antioxidant defenses, has shown otoprotective effects in three small randomized studies involving adults with end-stage renal failure receiving aminoglycosides, with pooled analysis suggesting a significant reduction in ototoxicity at 4–6 weeks (Kranzer et al., 2015). These studies were conducted in highly selected adult populations and were not designed to evaluate long-term auditory outcomes. Dedicated pediatric aminoglycoside otoprotection trials are lacking, and *in vitro* studies have demonstrated that NAC may antagonize the antimicrobial efficacy of gentamicin and tobramycin, a concern that requires resolution before routine clinical adoption (Rivetti et al., 2023). Aspirin has been evaluated in a prospective, randomized, double-blind trial of 195 patients receiving gentamicin, demonstrating a significant protective effect against ototoxicity (Sha et al., 2006; Chen et al., 2007). However, the applicability of these findings to neonatal and pediatric populations, where aspirin use carries specific risks, remains to be established. D-methionine, a direct and indirect antioxidant that augments cochlear mitochondrial GSH levels, has shown dose-dependent protection against gentamicin-, amikacin-, and tobramycin-induced ototoxicity in animal models without compromising aminoglycoside antimicrobial efficacy *in vitro* or *in vivo* (Fox et al., 2016). Human clinical trials of D-methionine for aminoglycoside otoprotection have not yet been reported.

Pharmacological modulation of intracellular trafficking has emerged as a preclinical research direction. Aminoglycosides sequestered in lysosomes may overwhelm lysosomal capacity, leading to lysosomal membrane permeabilization and cytosolic release of drug and hydrolases (Hailey et al., 2017). Autophagy enhancement with rapamycin or temsirolimus (mTOR inhibitors) has been shown to restore autophagic flux, upregulate Rab7-mediated autophagosome-lysosome fusion, reduce oxidative stress, and improve hair cell survival in cochlear explant and cell culture models of aminoglycoside ototoxicity (He et al., 2017; Kim et al., 2017; Ye et al., 2019). Impairment of PINK1-PRKN-mediated mitophagy has also been documented in neomycin-exposed hair cells, suggesting that mitochondrial quality control pathways are compromised during aminoglycoside injury (Zhang et al., 2023). These findings identify autophagy and intracellular trafficking as potential therapeutic targets, though none of these approaches have been evaluated in clinical studies.

Additional preclinical approaches under investigation include MET channel blockers that may prevent aminoglycoside entry into hair cells, intratympanic corticosteroid delivery to achieve high local anti-inflammatory concentrations, caspase and c-Jun N-terminal kinase (JNK) pathway inhibitors targeting apoptotic cell death cascades, and gene therapy strategies to enhance hair cell regeneration or resistance (Han et al., 2023). Translating these preclinical findings into clinical otoprotective strategies remains a major research priority, with the fundamental requirement that any candidate otoprotectant must not compromise aminoglycoside bactericidal efficacy. Given the exposure-dependent nature of aminoglycoside ototoxicity, pharmacokinetic optimization and duration minimization remain more immediately actionable strategies than adjunctive pharmacological otoprotection.

### Treatment duration optimization

Minimizing cumulative aminoglycoside exposure is among the most direct strategies for reducing ototoxic risk. In neonatal practice, aminoglycosides are most commonly prescribed as empiric combination therapy for suspected sepsis, with the expectation that treatment will be reassessed and narrowed within 48–72 h once culture results and clinical response are available (Cantey and Baird, 2017). De-escalation to targeted, non-ototoxic alternatives once pathogen identification and susceptibility data permit represents a core antimicrobial stewardship principle that simultaneously optimizes infection management and limits unnecessary ototoxic exposure (Klingenberg et al., 2018). When continued aminoglycoside therapy is clinically necessary, treatment duration should be guided by the specific indication, site of infection, and clinical response, with the shortest effective course preferred. Prolonged courses, particularly those exceeding 7–10 days or involving repeated treatment cycles, as in cystic fibrosis, carry substantially increased cumulative ototoxic risk. Observational and pharmacodynamic analyses suggest that risk increases nonlinearly with cumulative exposure, reinforcing the importance of early de-escalation and the integration of TDM, audiological monitoring, and stewardship-guided duration decisions as discussed in the preceding sections.

## Management of established ototoxicity

### Early identification and intervention planning

Even when exposure optimization strategies are employed, early identification of ototoxic injury remains essential, as cochlear damage may progress despite appropriate dosing and monitoring. When aminoglycoside-induced hearing loss is identified, whether through serial ototoxicity monitoring, universal newborn hearing screening, or clinical suspicion prompted by developmental concerns, comprehensive audiological characterization should follow promptly. The assessment modalities described in the Audiological Monitoring section serve both surveillance and diagnostic purposes. The typical audiometric configuration of aminoglycoside ototoxicity, a bilateral high-frequency sloping sensorineural loss, helps distinguish it from other etiologies, though asymmetric presentations occur and should not exclude the diagnosis (Landier, 2016). Although this configuration is typical, co-exposures and genetic susceptibility may modify the phenotype. Extended high-frequency audiometry is particularly valuable for confirming early cochlear injury that may not yet affect conversational frequencies. Vestibular assessment using vHIT, rotary chair testing, and VEMPs should be pursued in aminoglycoside-exposed children presenting with unexplained motor delays, coordination difficulties, or balance complaints, as pediatric vestibular dysfunction frequently presents through developmental rather than vertiginous symptoms and remains widely underdiagnosed (Rine and Wiener-Vacher, 2013).

The urgency of intervention reflects the dependence of speech, language, and cognitive development on early auditory input. For mild-to-moderate high-frequency losses, hearing aid fitting with frequency-specific amplification should be initiated as early as possible, guided by prescriptive targets validated for pediatric populations (American Academy of Audiology, 2013). When hearing loss is severe to profound, or progresses despite aminoglycoside discontinuation, early cochlear implantation should be considered, as discussed in the Hearing Rehabilitation section below; early intervention within the period of maximal auditory neuroplasticity is associated with optimal speech and language outcomes (Joint Committee on Infant Hearing, 2019). For children with confirmed vestibular hypofunction, vestibular rehabilitation therapy incorporating age-appropriate balance training and motor development support can improve functional outcomes (Rine et al., 2004). Early vestibular intervention may mitigate secondary delays in gross motor and spatial development. An integrated multidisciplinary approach encompassing audiology, otolaryngology, speech-language pathology, and developmental pediatrics ensures that rehabilitation addresses the full spectrum of communicative and motor consequences.

### Hearing rehabilitation

Hearing aid amplification is first-line rehabilitation for aminoglycoside-induced hearing loss. For the high-frequency sloping configuration typical of cochlear ototoxicity, frequency-specific amplification targeting the affected range can preserve access to speech cues critical for language development, while preserving residual low-frequency hearing. The Joint Committee on Infant Hearing 1–3–6 benchmarks (screening by 1 month, diagnosis by 3 months, and intervention by 6 months) provide a widely adopted framework for timely intervention, and aminoglycoside-exposed infants identified through serial monitoring should be referred into this pathway without delay (Joint Committee on Infant Hearing, 2019). Pediatric audiology expertise is essential, as real-ear verification, age-appropriate coupling, and ongoing adjustment as the child grows all influence the effectiveness of rehabilitation.

When amplification provides insufficient benefit, cochlear implantation offers effective rehabilitation for severe-to-profound aminoglycoside-induced losses. Outcomes are generally favorable because aminoglycoside ototoxicity is predominantly a sensory hair cell lesion, with relative preservation of spiral ganglion neurons and central auditory pathways, providing an intact neural substrate for electrical stimulation (Selimoglu, 2007). For children with preserved low-frequency hearing and disproportionately affected high frequencies, electroacoustic stimulation (EAS) combining electric and acoustic input may offer advantages, although pediatric data specific to aminoglycoside ototoxicity remain limited (Gantz et al., 2006). Regardless of the rehabilitation modality selected, early intervention within the period of maximal auditory neuroplasticity remains the strongest predictor of speech and language outcomes.

### Vestibular rehabilitation

Vestibular rehabilitation therapy promotes central compensation for peripheral vestibular deficits through structured exercise programs. Adaptation exercises improve vestibulo-ocular reflex gain through repetitive head movements with visual fixation, while substitution exercises develop alternative balance and gaze stability strategies using visual and proprioceptive cues (Herdman, 2013). In children, vestibular rehabilitation integrates with physical and occupational therapy to address motor development alongside vestibular function, though access to pediatric-specific vestibular rehabilitation remains limited, and evidence is drawn primarily from small case series (Rine et al., 2004). Home exercise programs adapted to the developmental stage, with parental involvement to support compliance, extend therapeutic reach. Among aminoglycosides, gentamicin and streptomycin pose the greatest vestibulotoxic risk; complete bilateral vestibular loss, though uncommon with contemporary dosing and monitoring, has been reported following prolonged or high cumulative exposure to gentamicin. Management of severe bilateral vestibular hypofunction focuses on maximizing residual function and strengthening compensatory strategies. Vestibular implants remain investigational and are currently limited to research settings (van de Berg et al., 2020).

### Ongoing care and family-centered support

Long-term audiological surveillance is essential, as aminoglycosides accumulate within cochlear hair cells and may contribute to delayed or progressive dysfunction even after treatment discontinuation (Wang and Steyger, 2009). Audiometric reassessment at least annually for several years following exposure, with increased frequency if progression is detected, is consistent with current ototoxicity monitoring frameworks (Garinis et al., 2021). Children with confirmed hearing loss benefit from early intervention services addressing speech, language, and communication development, with the choice of communication approach (auditory-verbal, cued speech, or sign language) guided by family preferences, hearing loss severity, and available resources. Educational accommodations, including remote microphone technology, acoustic classroom modifications, and individualized education programs, support academic access.

Family education about etiology, prognosis, and intervention options empowers informed decision-making. When MT-RNR1 or other mitochondrial variants have been identified, genetic counseling provides essential information for family planning and cascade testing of maternal relatives, as discussed in the Genetic Susceptibility section above. Psychological support addresses the emotional impact of iatrogenic hearing loss, which may carry a substantial burden for families. Parental grief following diagnosis of childhood hearing loss is often chronic and may be compounded by feelings of guilt or anger when the etiology is treatment-related, requiring sensitive, ongoing support (Kurtzer-White and Luterman, 2003). Medical alert documentation ensures that future prescribers are aware of ototoxicity history, enabling informed decisions about aminoglycoside re-exposure. Children and families should be advised that lifetime aminoglycoside avoidance is recommended when alternatives are available, and that enhanced monitoring is essential when re-exposure is clinically unavoidable.

## Discussion

The past two decades have substantially advanced understanding of aminoglycoside ototoxicity from hair cell entry mechanisms and intracellular trafficking to genetic susceptibility and exposure-response relationships. Pharmacokinetic optimization strategies, otoprotective candidates, and point-of-care genetic screening have all progressed from concept to clinical feasibility. Yet a persistent gap remains between available evidence and routine practice: most neonatal units do not screen for MT-RNR1 variants, extended-interval dosing adoption remains uneven, and model-informed dosing is largely confined to specialist centers.

### Controversies and schools of thought

The case for pre-treatment genetic screening is compelling. Point-of-care assays can identify MT-RNR1 carriers within approximately 30 min, and implementation data from a United Kingdom pragmatic trial support routine NICU screening within health systems where rapid genotyping infrastructure is available, without disruption to clinical workflows or time to antibiotics (McDermott et al., 2022a). Despite this evidence, screening has not yet become standard of care. Barriers include test availability, workflow integration, uncertainty regarding alternative antibiotic regimens, and the complexity of managing incidental findings from genetic testing.

Extended-interval dosing has achieved broader adoption. Meta-analytic data demonstrate equivalent efficacy and reduced nephrotoxicity with extended-interval regimens, with a non-significant trend toward lower ototoxicity in some analyses (Contopoulos-Ioannidis et al., 2004; Barza et al., 1996). However, neonatal implementation has been slower, reflecting the substantial pharmacokinetic variability characteristic of preterm and critically ill neonates. Without dosing nomograms validated across the full range of gestational ages and clinical contexts, clinicians may be reluctant to adopt unfamiliar extended-interval dosing regimens. Antimicrobial stewardship practices, including systematic reassessment at 48–72 h and de-escalation when cultures permit (Cantey and Baird, 2017), offer a complementary strategy for limiting cumulative exposure, though institutional compliance varies.

Therapeutic drug monitoring practice varies considerably across institutions. Some units rely on peak-and-trough concentrations, while others have adopted Bayesian dosing software. AUC-targeted monitoring may offer more direct alignment with cumulative exposure metrics implicated in ototoxic risk but requires additional sampling and dedicated analytical infrastructure. Comparative trials evaluating the relative ototoxicity-predictive value of these approaches in neonatal populations are lacking; until such data emerge, standardization will remain difficult. Similarly, although loop diuretic and aminoglycoside co-administration is common in critically ill neonates, strategies to minimize combined ototoxic exposure, including staggering administration timing and considering alternative diuretics, lack robust evidence but represent reasonable clinical practice (Lanvers-Kaminsky and Ciarimboli, 2017; Bates et al., 2002).

The relative contributions of genetic and environmental risk factors remain incompletely defined. MT-RNR1 variants confer high individual risk but explain only a minority of cases at the population level (McDermott et al., 2022b). Nuclear genetic variants in *TRMU*, *TFB1M*, *MTO1*, and *GTPBP3* may modify the penetrance of MT-RNR1-associated ototoxicity (Gao et al., 2017; Bykhovskaya et al., 2004), but associations require validation in adequately powered pediatric cohorts before clinical application. When children have identified risk factors but no confirmed genetic susceptibility, clinicians face difficult trade-offs. Should alternative antibiotics be pursued aggressively, potentially compromising efficacy for some infections? Is intensified monitoring with standard aminoglycoside doses sufficient? These decisions must be individualized based on infection severity, available alternatives, and informed family preferences.

### Challenges in pediatric populations

Neonates pose unique challenges for the prevention and detection of ototoxicity. Developmental pharmacokinetic variability necessitates age-adjusted dosing and monitoring strategies that account for rapidly changing renal function. Audiological assessment relies primarily on objective measures that may miss subtle frequency-specific losses; the limitations of OAE and ABR in detecting early high-frequency ototoxicity underscore the need for age-appropriate behavioral and extended high-frequency testing as developmental stage permits, as discussed in the Audiological Monitoring section above. More broadly, the developmental impact of subclinical hearing loss insufficient to trigger screening failure but potentially affecting speech and language acquisition remains inadequately characterized. Validated pediatric vestibular screening protocols are also lacking, and vestibular function is tightly linked to early motor and cognitive development; symptoms are difficult to elicit, often presenting as motor delay or imbalance rather than vertigo, strengthening the case for age-appropriate screening and referral pathways (Wiener-Vacher et al., 2018; Wiener-Vacher et al., 2013; Rine et al., 2004; Rine and Wiener-Vacher, 2013).

### Research gaps and future directions

Several areas require focused investigation. Pediatric-specific trials of otoprotective agents should build on promising preclinical and adult data. To date, no randomized controlled trial has evaluated NAC, D-methionine, or aspirin for aminoglycoside otoprotection specifically in neonates or children. Existing human evidence is confined to selected adult populations (NAC and aspirin), while pediatric translation remains uncertain (Kranzer et al., 2015; Sha et al., 2006; Chen et al., 2007). A key prerequisite for pediatric trials is demonstrating that there is no clinically meaningful reduction in aminoglycoside bactericidal activity. For NAC, some studies have raised concern about the potential antagonism of gentamicin and tobramycin (Kranzer et al., 2015), whereas D-methionine has shown otoprotection without antimicrobial interference in animal models (Fox et al., 2016).

Biomarkers enabling early detection of cochlear injury before irreversible threshold shifts would significantly advance clinical practice. Prestin has shown promise as a serological biomarker in ototoxicity models, with elevations preceding ABR threshold shifts in cisplatin-exposed animals and dose-related increases reported in amikacin-exposed models (Naples et al., 2018; Dogan et al., 2018). However, prestin-knockout control work has raised concerns about assay specificity and reliability using current ELISA-based approaches (Zheng et al., 2024). No studies have yet evaluated prestin or other candidate inner-ear protein biomarkers specifically in the context of neonatal aminoglycoside exposure, and this remains a clear translational gap (Rivetti et al., 2023).

Elucidating the mechanisms underlying delayed-onset and progressive ototoxicity, including ongoing oxidative stress, mitochondrial dysfunction, and sustained inflammatory signaling following exposure, may identify additional therapeutic targets and inform the optimal duration and intensity of post-treatment audiological surveillance (Bottger and Schacht, 2013; Mukherjea et al., 2011; Ruhl et al., 2019; Han et al., 2023).

In the longer term, advances in hair cell regeneration and gene therapy may fundamentally change the management landscape. Mammalian cochlear hair cells do not spontaneously regenerate (Forge and Li, 2000), but reprogramming studies using transcription factor combinations (including Atoh1 with Gfi1 and Pou4f3) have generated hair cell-like cells in adult cochlear tissue, with partial maturation and evidence of neural attraction (McGovern et al., 2024). Separately, AAV-based gene therapies for monogenic hearing loss have entered clinical trials, with early pediatric otoferlin (OTOF) replacement data showing hearing improvement in most treated children (Lv et al., 2024). While these approaches currently target genetic rather than acquired hearing loss, inner-ear vector delivery and cell fate conversion technologies may become relevant to drug-induced ototoxicity if reliable regeneration can be achieved (Han et al., 2023).

Pharmacogenomic equity represents another important gap. Carrier-frequency data for MT-RNR1 variants are best characterized in European cohorts, with fewer population-level estimates elsewhere. Expanding prevalence datasets across diverse populations, including East Asian and African estimates already reported, and developing affordable point-of-care testing suitable for low-resource settings are priorities if screening is to be globally equitable (McDermott et al., 2022a; Vandebona et al., 2009; Bitner-Glindzicz et al., 2009; Gaafar et al., 2024; NICE, 2023; Lu et al., 2010; Wu et al., 2007).

Implementation science research should identify strategies for translating evidence into routine care across diverse healthcare settings. The most immediate opportunity to reduce the burden of aminoglycoside ototoxicity in children lies in closing the implementation gap across genetic screening, dosing optimization, monitoring protocols, and rehabilitation access.

### Clinical implications

Current evidence supports the following practice recommendations: implement MT-RNR1 screening in NICU settings where rapid testing infrastructure is available (McDermott et al., 2022a; McDermott et al., 2022b). Use extended-interval dosing with age-appropriate protocols (Contopoulos-Ioannidis et al., 2004; Nestaas et al., 2005; Rao et al., 2016). Monitor serum concentrations to prevent accumulation, using trough-based monitoring as a minimum standard and AUC-guided approaches where feasible (Yu et al., 2020; Hartman et al., 2020; Keizer et al., 2018). Minimize concomitant ototoxin exposure and keep treatment courses short. Incorporate structured auditory (Table 5) and vestibular monitoring (Table 6, particularly following gentamicin exposure) (American Academy of Audiology, 2009; Garinis et al., 2021). When ototoxicity is identified, initiate rehabilitation without delay (American Academy of Audiology, 2013).

Despite optimal management, some children will sustain permanent hearing loss. However, rehabilitative outcomes have improved substantially. Current digital hearing aid technology provides effective amplification across a wide frequency range (American Academy of Audiology, 2013), cochlear implantation achieves good outcomes when candidacy criteria are met (Gantz et al., 2006), and the critical importance of intervention within the first 6 months of identification is now well established (Joint Committee on Infant Hearing, 2019). The trajectory for a child diagnosed with aminoglycoside-induced hearing loss today is markedly better than a generation ago.

## Conclusions

The tools to reduce aminoglycoside ototoxicity in neonates and children already exist. Rapid point-of-care genotyping can identify MT-RNR1 carriers within 30 min. Extended-interval dosing and Bayesian-guided pharmacokinetic optimization can minimize toxic exposure while preserving efficacy. Concomitant ototoxin exposure can be reduced through judicious prescribing, and cumulative aminoglycoside exposure limited through antimicrobial stewardship and abbreviated treatment courses, with structured audiological and vestibular surveillance maintained throughout and beyond treatment. The challenge is no longer one of evidence but of implementation. Closing the gap between what is known and what is routinely practiced in genetic screening, dosing optimization, monitoring, and rehabilitation access represents the most immediate opportunity to prevent irreversible hearing loss in the children most vulnerable to it.

## Glossary

4-HNE: 4-hydroxynonenal
ABR: auditory brainstem response
ALSPAC: Avon Longitudinal Study of Parents and Children
AUC: area under the curve
AUROC: area under the receiver operating characteristic curve
**Ca** ^**2+**^: calcium ion
**casp** ^**3**^: caspase-3
**casp** ^**9**^: caspase-9
CDSS: clinical decision support system
CI: confidence interval
CPIC: Clinical Pharmacogenetics Implementation Consortium
DPOAE: distortion product otoacoustic emission
EAS: electroacoustic stimulation
ER: endoplasmic reticulum
GFR: glomerular filtration rate
GSH: glutathione
GSSG: glutathione disulfide
ICH: International Council for Harmonisation
IHC: inner hair cell
JNK: c-Jun N-terminal kinase
LBW: low birth weight
MDR-TB: multidrug-resistant tuberculosis
MET: mechanoelectrical transduction
MIPD: model-informed precision dosing
MLKL: mixed lineage kinase domain-like protein
MT-RNR1: mitochondrially encoded 12S ribosomal RNA
MTO1: mitochondrial tRNA translation optimization 1
NAC: N-acetylcysteine
NICE: National Institute for Health and Care Excellence
NICU: neonatal intensive care unit
NOX3: NADPH oxidase 3 (NOX3)-dependent superoxide production
OAE: otoacoustic emissions
OHC: outer hair cell
OR: odds ratio
PALOH: Pharmacogenetics to Avoid Loss of Hearing
PBPK: physiologically based pharmacokinetic
RIPK: receptor-interacting protein kinase
ROS: reactive oxygen species
RR: relative risk
SCC: semicircular canal
TDM: therapeutic drug monitoring
TFB1M: transcription factor B1, mitochondrial
TRMU: tRNA 5-methylaminomethyl-2-thiouridylate methyltransferase
TRP: transient receptor potential
TUNEL: terminal deoxynucleotidyl transferase dUTP nick end labeling
VEMP: vestibular evoked myogenic potential
vHIT: video head impulse test
